# Twelve Months of Routine HIV Screening in 6 Emergency Departments in the Paris Area: Results from the ANRS URDEP Study

**DOI:** 10.1371/journal.pone.0046437

**Published:** 2012-10-02

**Authors:** Enrique Casalino, Bruno Bernot, Olivier Bouchaud, Chakib Alloui, Christophe Choquet, Elisabeth Bouvet, Florence Damond, Sandra Firmin, Aurore Delobelle, Beatrice Ename Nkoumazok, Guillaume Der Sahakian, Jean-Paul Viard, Olivier Zak Dit Zbar, Elisabeth Aslangul, Anne Krivine, Julie Zundel, Jade Ghosn, Patrice Nordmann, Yann-Erick Claessens, Tassadit Tahi, Bruno Riou, Agnès Gautheret-Dejean, Christine Katlama, Pierre Hausfater, Françoise Brun-Vézinet, Dominique Costagliola

**Affiliations:** 1 AP-HP, Groupe Hospitalier Universitaire Paris Nord-Val de Seine, Service d’accueil des Urgences, Paris, France; 2 Université Denis-Diderot Paris 7, Paris, France; 3 AP-HP, Hôpitaux Universitaire Paris Seine St Denis, CHU Avicenne, Service d’accueil des Urgences, Bobigny, France; 4 AP-HP, Hôpitaux Universitaire Paris Seine St Denis, CHU Avicenne, Service des Maladies Infectieuses et Tropicales, Bobigny, France; 5 Université Paris 13, Bobigny, France; 6 AP-HP, Hôpitaux Universitaire Paris Seine St Denis, CHU Avicenne, Laboratoire de virologie, Bobigny, France; 7 AP-HP, Groupe Hospitalier Universitaire Paris Nord-Val de Seine, Service des maladies infectieuses et tropicales, Paris, France; 8 AP-HP, Groupe Hospitalier Universitaire Paris Nord-Val de Seine, Laboratoire de Virologie, Paris, France; 9 Université Denis-Diderot Paris 7, EA 4409, Paris, France; 10 INSERM U943, Paris, France; 11 UPMC Univ Paris 06 UMR S943, Paris, France; 12 AP-HP, Hôpital Hôtel-Dieu, Service d’accueil des Urgences/SMUR, Paris, France; 13 Université Paris Descartes Paris 5, Paris, France; 14 AP-HP, Hôpital Hôtel-Dieu, Centre de Diagnostic et Thérapeutique, Paris, France; 15 Université Paris Descartes Paris 5, EA 3620, Paris, France; 16 AP-HP, Hôpital Hôtel-Dieu, Service de Pneumologie, Paris, France; 17 AP-HP, Hôpital Hôtel-Dieu, Service de Médecine Interne, Paris, France; 18 Université Paris Descartes Paris 5, Paris, France; 19 AP-HP, Hôpital Cochin, Laboratoire de virologie, Paris, France; 20 AP-HP, Hôpital Bicêtre, Service d’accueil des Urgences, Le Kremlin Bicêtre, France; 21 AP-HP, Groupe Hospitalier Bicêtre, Service de medicine interne et maladies infectieuses, Le Kremlin Bicêtre, France; 22 AP-HP, Groupe Hospitalier Bicêtre, Laboratoire de bactério-virologie, Le Kremlin Bicêtre, France; 23 Université Paris-Sud UMR S914, Le Kremlin Bicêtre, France,; 24 INSERM U914, Le Kremlin Bicêtre, France; 25 AP-HP, Hôpital Cochin, Service d’accueil des Urgences, Paris, France; 26 Université Paris Descartes Paris 5, Paris, France; 27 AP-HP, Hôpital Cochin, Service de médecine interne et des maladies infectieuses, Paris, France; 28 AP-HP, Groupe Hospitalier Pitié-Salpêtrière, Service d’accueil des urgences, Paris, France; 29 UPMC Univ Paris 06, Paris, France; 30 APHP, Groupe Hospitalier Pitié-Salpêtrière, Laboratoire de Virologie, Paris, France; 31 AP-HP, Groupe Hospitalier Pitié-Salpêtrière, Service de Maladies Infectieuses, Paris, France; Rush University, United States of America

## Abstract

**Objective:**

In October 2009 the French National Authority for Health recommended that HIV testing be proposed at least once to all persons aged 15 to 70 years in all healthcare settings. We examined whether routine HIV screening with a rapid test in emergency departments (EDs) was feasible without dedicated staff, and whether newly diagnosed persons could be linked to care.

**Methods:**

This one-year study started in December 2009 in 6 EDs in the Paris area, using the INSTI™ test. Eligible individuals were persons 18 to 70 years old who did not present for a vital emergency, for blood or sexual HIV exposure, or for HIV screening. Written informed consent was required.

**Results:**

Among 183 957 eligible persons, 11 401 were offered HIV testing (6.2%), of whom 7936 accepted (69.6%) and 7215 (90.9%) were tested (overall screening rate 3.9%); 1857 non eligible persons were also tested. Fifty-five new diagnoses of HIV infection were confirmed by Western blot (0.61% (95% CI 0.46–0.79). There was one false-positive rapid test result. Among the newly diagnosed persons, 48 (87%) were linked to care, of whom 36 were not lost to follow-up at month 6 (75%); median CD4 cell count was 241/mm^3^ (IQR: 52–423/mm^3^).

**Conclusions:**

Screening rates were similar to those reported in opt-in studies with no dedicated staff. The rate of new diagnoses was similar to that observed in free anonymous test centres in the Paris area, and well above the prevalence (0.1%) at which testing has been shown to be cost-effective.

## Introduction

In most industrialized countries about one-third of HIV-infected patients access the health care system with advanced HIV disease (CD4 cell count below 200/mm2 or AIDS), and one-half late (CD4 cell count below 350/mm^3^ or AIDS) [1]–[4]. Late presentation is associated with an excess risk of death [1], [4], [5] and with higher costs [6], [7]. Several studies have shown that expanded screening is cost-effective if the prevalence of undiagnosed HIV infection is above 0.1% [8], [9]. As a result, many countries have recently issued new testing guidelines [10]–[12].

In France, the National Authority for Health issued new guidelines on HIV screening in 2009: systematic HIV screening was to be proposed at least once to all people aged from 15 to 70 years, regardless of signs or symptoms, and whatever the risk profile, in addition to targeted HIV screening every year for men who have sex with men (MSM), intravenous drug users (IDUs), and heterosexuals from sub-Saharan Africa and the Caribbean region who have multiple partners. An estimated 25 000 to 30 000 persons in France have undiagnosed HIV infection (roughly 7 per 10 000 inhabitants) [13]. The Paris area is one of the most affected regions of France, accounting for 44% of new diagnoses [14], and with an estimated prevalence of undiagnosed infection slightly above 0.1%.

Several studies, mainly conducted in the USA, have evaluated the feasibility of expanded HIV screening based on the use of rapid tests in emergency departments [15]–[26], with or without dedicated staff, and with opt-in or opt-out consent. They showed that the unknown prevalence was above the cost-effectiveness threshold of 0.1%. Screening rates ranged from 1.8% to 65.2%. Results may be different in Europe where health care systems are completely different.

In France, public hospital emergency departments (EDs) receive 15 to 17 million visits every year, representing about 20% to 25% of the population [27]. As such, they appear suited to non-targeted HIV screening. Use of HIV rapid tests at points of care could permit new HIV testing strategies. On the other hand, EDs are crowded with increasing number of passage over the recent years, with a 2.5–3.5% rate of increase per year in Paris area [27]. Here we described routine HIV screening, without extra staff, using a rapid test in 6 Paris region EDs and whether persons with newly diagnosed HIV infection could be linked to care.

## Methods

### Study Objectives

The primary objective of the ANRS URDEP study (URDEP stands for URgences and DEPistage, i.e. Emergency and Screening) was to describe the outcomes of routine HIV screening with a rapid test in 6 adult EDs in the Paris area, with no additional staff. Secondary objectives were to determine the proportion of ED attendees who were offered screening, the acceptance rate, the rate of screening, the number of new diagnoses, and the numbers and characteristics of newly diagnosed individuals who were linked to care, both initially and for a six-month period. Factors influencing test proposal and test positivity were also evaluated. We were unable to conduct a routine opt-out study, as the use of rapid tests in this context was not authorised at the time: we were thus obliged to undertake a research project with the participants’ written informed consent (opt-in).

### Study Centres and Subjects

The study lasted 12 months and took place in 6 teaching-hospital EDs in the Paris area. It began between December 2009 and March 2010, depending on the centre. Eligible persons were subjects 18 to 70 years old consulting the ED, whose HIV serostatus was unknown or who had a negative HIV serology dating back more than 6 months, and who gave their written informed consent. Persons whose clinical status was incompatible with the expression of consent (altered consciousness, severe psychiatric disorders, short-term or medium-term vital emergency), and persons known to be HIV-seropositive were not eligible. We secondarily excluded persons presenting because of blood or sexual HIV exposure or for HIV screening. The study was approved by the Saint Germain en Laye Institutional Review Board.

### Screening Procedures

**Figure 1:**
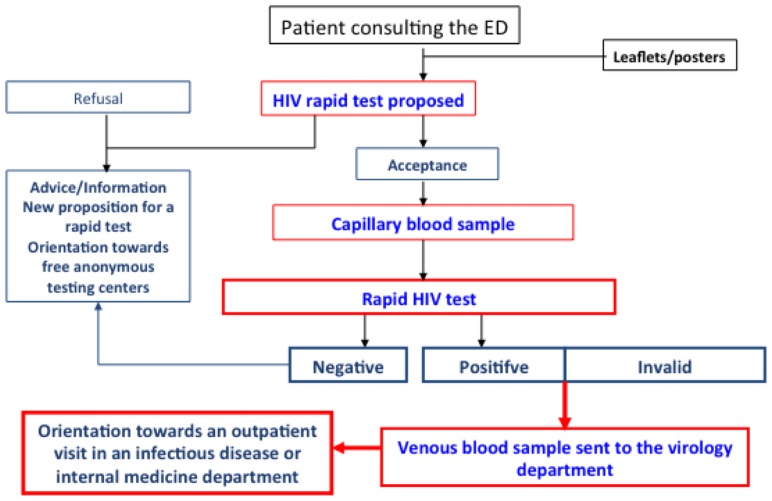
Flow-chart of procedures for HIV screening with a rapid test in the ANRS URDEP study.

Before the beginning of the study, ED staff members (nurses or physicians) were trained in how to inform, propose and perform the rapid test, and to counsel. Figure 1 depicts the procedure. All the EDs displayed posters and brochures in their waiting rooms and registration areas, advertising the availability of free rapid HIV screening. Patients could ask to participate to the study and ED staff members, whether triage or other nurses, senior physicians or interns, could offer testing to eligible persons on any day of the week, at any time, as well as obtain written consent, provide pre-test information, and administer the HIV test, in addition to their usual responsibilities. Patients with negative results were informed by the person who performed the test, and were given written information on HIV prevention. Positive and invalid results were given by senior physicians only. All tested patients also received a written result, dated and signed. No additional staff were provided for the study.

A delocalized biology station was established in each ED with a register to record the performed tests, under the supervision of the virology laboratory of each participating hospital. The INSTI™ HIV-1/HIV-2 Rapid Antibody Test (BioLytical Laboratories, Richmond British Columbia, Canada, Nephrotek France) was used. This FDA- and EC-approved test can be performed on 50 µl of finger-stick capillary blood, and provides the result in 5 minutes with few manipulations. The test detects antibodies to both HIV-1 and HIV-2 (test spot) and also human non-specific IgG antibodies (control spot). It is invalid when the control spot is negative. A confirmatory venous blood sample had to be obtained in case of a positive or invalid result. An appointment had to be organized with a specialist in HIV/AIDS within 72 hours of all positive rapid tests. Persons who attended this appointment were considered linked to care and were followed for six months.

### Statistical Considerations

Age, gender and test proposal, acceptance/refusal and results were collected for each patient on the ED computer system. The subject’s geographic origin, transmission group, history of HIV testing, CD4 cell count, and AIDS status were also collected for newly diagnosed persons linked to care. Logistic regression was used to identify factors associated with test proposal to eligible persons, and with new diagnosis of HIV seropositivity among eligible tested persons. All tests were two-sided, and significance was assumed at p<0.05. SAS software version 9.3 and STATA 12 were used for all analyses. As this was a feasibility study, no formal sample size calculation was performed. The study duration was 12 months, to account for the variability of ED activity over the year.

## Results

**Figure 2:**
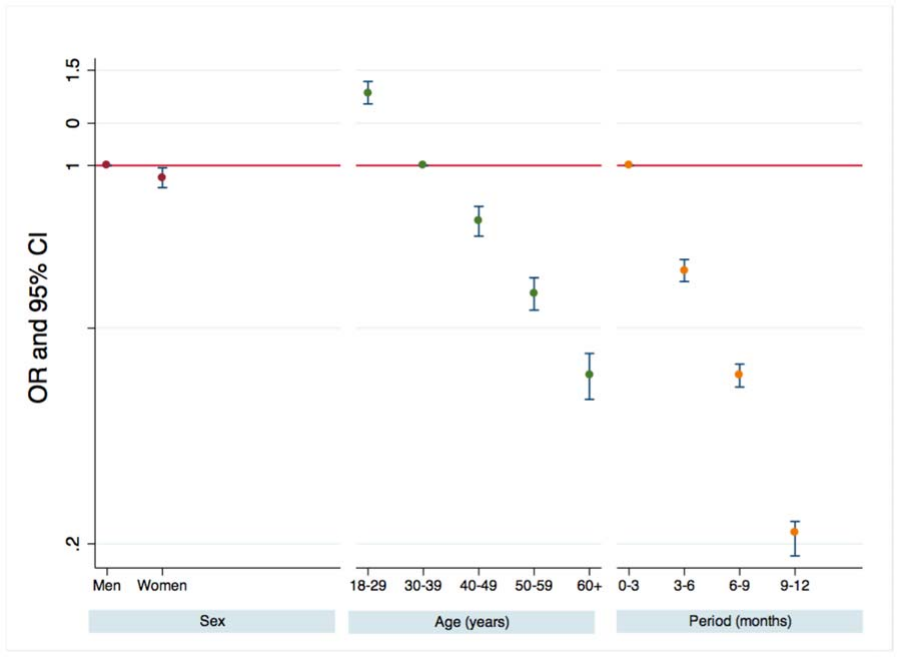
Factors associated with test proposal to eligible persons.

During the 12-month study period, 311 153 persons presented to the 6 ED departments, of whom 183 957 were eligible and 127 196 non-eligible, including 1959 persons who presented for HIV sexual or blood exposure or for HIV screening (Table 1 and Table S1). Among the eligible persons (Table 1), 11 401 were offered the rapid test (6.2% overall; 2.6% to 10.7% according the ED; P<0.001). Multivariable analyses stratified on the centre (Figure 2) showed that women were slightly less likely than men to be offered the test (6.0% versus 6.3%, p = 0.012). Younger persons were far more likely to be offered the test (8.3% between 18 and 29 years, 2.9% of persons 60 or over). The rate of test proposal fell from 10.6% during the first 3 months to 2.5% during the last 3 months. The same trend was observed in all the centres.

**Table 1:** Results of a 12 month routine HIV screening program with a rapid test after written inform consent in eligible patients at 6 university hospital emergency departments (EDs) in Paris area (2009–2011).

Centre	A	B	C	D	E	F	TOTAL
**Persons examined at EDs**	51111	44611	68234	42525	68511	36161	311153
**Eligible persons**	30284	24872	28182	28499	45178	26942	183957
**Offered HIV testing**	782	1085	1291	2537	2815	2891	11401
	(2.6%)	(4.4%)	(4.6%)	(8.9%)	(6.2%)	(10.7%)	(6.2%)
**Accepting HIV testing**	633	789	1032	1370	1955	2157	7936
	(80.9%)	(72.7%)	(79.9%)	(54.0%)	(69.4%)	(74.6%)	(69.6%)
**Tested for HIV**	617	567	988	983	1917	2143	7215
	(97.5%)	(71.9%)	(95.7%)	(71.8%)	(98.1%)	(99.4%)	(90.9%)
**Rate tested for HIV among ED** **patients**	2.0%	2.3%	3.5%	3.5%	4.2%	8.0%	3.9%
**Positive rapid test**	6	3	11	5	13	12	50
**Positive test in previously unknown** **HIV-infected patients**	5	3	9	5	11	11	44
**Western-blot confirmatory test performed**	5	3	9	4	10	10	41
	(100%)	(100%)	(100%)	(80.0%)	(91%)	(91%)	(93%)
**Newly identified HIV infected patients**	5	3	8	4	10	10	40
**Rate of newly identified HIV-infected patients among tested eligible patients**	0.81%	0.53%	0.81%	0.41%	0.52%	0.47%	0.55%
**Linked to care**	5	2	7	4	9	10	37
	(100%)	(67%°	(87%)	(100%)	(90%)	(100%)	(92.5%)
**Not lost to follow-up at month 6**	4	2	4	3	5	10	28
	(80%)	(100%)	(57%)	(75%°	(56%)	(100%)	(76%)

**Figure 3:**
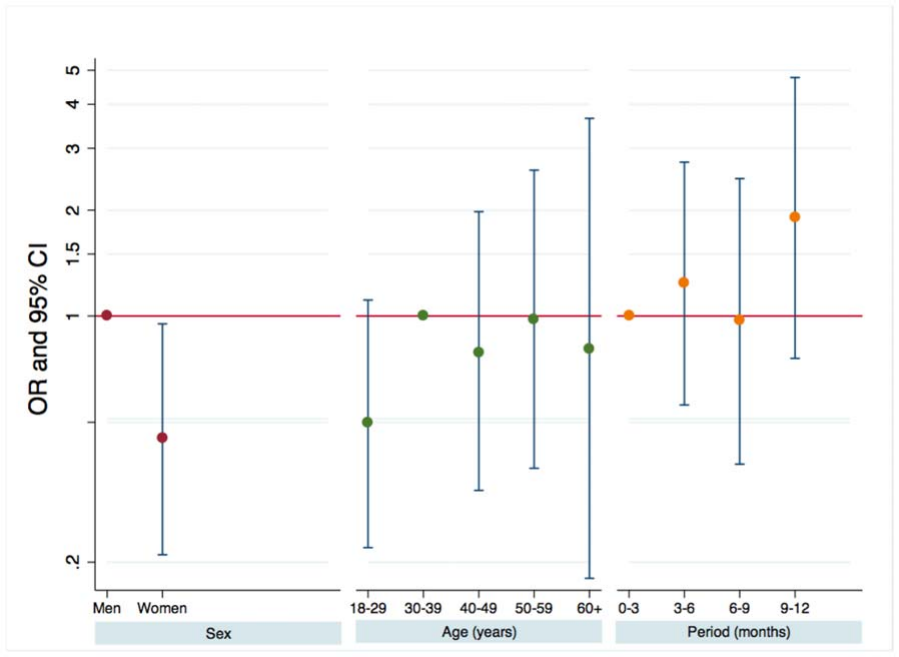
Factors associated with being newly diagnosed HIV-seropositive among eligible tested persons.

Test acceptance rates ranged from 54.0% to 80.9% depending on the centre. Overall, 7936 persons (69.6%) accepted to be tested, of whom 7215 (90.9% of those who accepted and 3.9% of all eligible ED attendees) were tested. For the remaining 761 persons, the test was not performed for various reasons: the staff forgot to perform the test, the person leaved the ED before being tested, … Fifty persons had a positive result, of whom 6 acknowledged only at this stage that they already knew their HIV serostatus. Forty-one of the remaining 44 persons had a confirmatory Western blot, and all but one were confirmed as newly diagnosed. Finally, the estimated rate of new diagnoses was 0.55% (95% confidence interval (95% CI): 0.40–0.75%), with no difference according to the centre (p = 0.527). In multivariable analyses stratified on the centre (Figure 3), women were less likely than men to be newly diagnosed as HIV-seropositive (0.32% versus 0.71%, p = 0.0371). Neither age nor the study period (in three-month periods) was associated with the likelihood of being diagnosed as HIV-seropositive.

The rapid test was also offered to 2520 non eligible persons (Table S1), i.e. persons younger or older than the age range specified by the protocol, or with a medium-term vital emergency, or presenting for HIV blood or sexual exposure or HIV screening. This represented 2.0% of non-eligible ED attendees overall, and from 0.6% to 6.7% according to the centre. Centre D, located in a gay quarter, accounted for 37% of all tests offered to non-eligible persons. Acceptance rates were high in all the centres (81.9%), and 1857 of the 2063 persons who accepted (with their written inform consent) were tested (90.0% of those accepting and 1.5% of all non-eligible ED persons). Of these, 23 were positive, of whom 3 already knew they were HIV-seropositive. Fifteen of these persons had a confirmatory Western blot and all were positive. Thus, the estimated rate of new diagnoses among non-eligible persons was 0.81% (95% CI: 0.45–1.33%), a rate not significantly different from that observed among eligible persons (p = 0.210).

**Table 2:** Characteristics of newly identified HIV infected individuals linked to care according to whether they were eligible or not.

	Eligible patients	Non eligible patients
	(n = 37)	(n = 11)
Age (years)		
< = 30	12	5
30–50	19	5
>50	6	1
Sex, origin and transmission group		
Men who have sex with men	13	6
Heterosexual woman of sub-Saharan origin	6	1
Heterosexual man of sub-Saharan origin	14	1
Heterosexual woman of other origin	2	1
Heterosexual man of other origin	2	1
IV drug user woman of other origin	-	1
Disease stage		
CD4 cell count < = 200/mm^3^ or AIDS	17	4
CD4 cell count 200–350/mm^3^	3	2
CD4 cell count >350/mm^3^	17	4
Unknown	-	1
Median CD4 cell count (interquartile range)	232 (52–406)	260 (142–423)
Previous HIV testing		
No	18	3
Yes, more than 2 years ago	11	5
Yes, in the past 2 years	7	2
Unknown	1	1

The estimated overall rate of new diagnoses among all tested ED attendees was 0.61% (95% CI: 0.46–0.79). Regardless of eligibility and the centre, the prevalence of previously undiagnosed HIV infection was always well above 0.1%.

The person with a false-positive rapid test was negative by Western blot, in two antigen-antibody combined tests, and in p24 antigen assays (one p24 antigen test and two antigen-antibody combined tests). Among the 9072 tests performed, 28 gave an invalid result (0.31%, 95% CI: 0.21–0.45%). In 19 of these cases, a second rapid test was performed or a venous blood sample was taken for standard antibody testing. All were negative.

**Table 3:** Summary of studies reporting non targeted HIV screening program in emergency departments, using rapid test since 2006.

First author orSource	Publicationyear	Dedicatedstaff	Consentapproach	Numbereligible	Numbertested	Screeningrate	Prevalencerate
MMWR NY	2007	No	Opt-in	72,948	1,288	0.018	0.015
MMWR La	2007	No	Opt-in	47,736	1,700	0.036	0.008
MMWR OAK	2007	Yes	Opt-in	66,731	6,368	0.095	0.010
Brown	2007	Yes	Opt-out	13,240	2,486	0.188	0.004
Lyss	2007	Yes	Opt-in	4,849	2,824	0.582	0.012
Silva	2007	Yes	Opt-in	3,030	1,428	0.471	0.006
Walenski	2008	Yes	Opt-in	2,356	854	0.362	0.006
White	2009	No	Opt-in	11,8324	7,923	0.067	0.007
Haukoos	2010	No	Opt-out	28,043	6,933	0.247	0.002
Torres (F)	2011	No	Opt-in	52,542	3,623	0.069	0.009
Torres (C)	2011	Yes	Opt-in	27,913	1,300	0.047	0.008
White	2011	Yes	Opt-in	23,236	4,053	0.174	0.002
White	2011	Yes	Opt-out	26,757	4,679	0.175	0.004
Sattin	2011	Yes	Opt-out	13,035	8,504	0.652	0.004
D’Almeida	2012	Yes	Opt-in	78,411	12,754	0.163	0.001
**Combined results of the studies**							
No dedicated staff, opt-in		No	Opt-in	291,550	14,534	0.050	
Dedicated staff, opt-in		Yes	Opt-in	206,526	29,581	0.143	
No dedicated staff, opt-out		No	Opt-out	28,043	6,933	0.247	
Dedicated staff, opt-out		Yes	Opt-out	53,032	15,669	0.295	

Out of the 55 newly diagnosed persons, 48 were linked to care (87% overall, 95% CI: 76–95%; 92.5% and 73% of eligible and non-eligible persons, respectively; p = 0.079), and 36 (75%; two-thirds of all new diagnoses) were still in care at month 6. The characteristics of the 48 persons linked to care are shown in Table 2. There was no difference between eligible and non-eligible persons. Thirty-seven (77%) of the newly diagnosed HIV-infected persons linked to care were men, 19 (40%) were MSM, 22 (46%) were from sub-Saharan Africa, 17 (35%) were 30 years of age or younger, and 7 (15%) were over 50 years old. Twenty-six persons (54%) were not at an advanced stage of HIV disease (no prior AIDS events, and CD4 cell counts >200/mm^3^), and the median CD4 cell count was 241/mm^3^ (interquartile range: 52–423/mm^3^). The prior test history was known for 46 of the 48 persons: 21 (44%) had not been tested before, 16 (33%) had been tested more than 2 years previously, and 9 (19%) had been tested in the previous 2 years. Among the 25 persons who were diagnosed late (with AIDS or a CD4 cell count ≤350/mm^3^), 15 (60%) had not been tested before, compared to 29% (6/21) of those not presenting late (p = 0.042).

## Discussion

We were able to test for HIV infection 3.9% of eligible persons presenting to 6 emergency departments in the Paris area, representing one-third of all ED attendees in this area, with no additional staff and with obligatory written informed consent (opt-in approach). The test rate varied widely across the six centres (from 2.0% to 8.0%) and during the one-year study period (from 10.6% during the first 3 months to 2.5% during the last 3 months). The prevalence of previously undiagnosed HIV infection was 0.55% (95% CI: 0.40–0.75) among eligible persons, a rate well above the reported cost-effectiveness threshold of 0.1% [8], [9]. The new diagnoses involved MSM or persons originating from sub-Saharan Africa in 85% of cases. The median CD4 cell count at diagnosis was 241/mm^3^, and 44% of newly diagnosed persons had never previously been tested for HIV. Two-thirds of the newly diagnosed persons were durably linked to care.

In a context of diminishing financial and human resources, a screening study with no additional staff mimicked real-life implementation of routine HIV screening in emergency departments. Because of contemporary French regulations, this study had to take the form of a research project with written inform consent (opt-in approach). An opt-out strategy without written consent is recommended in most settings, and would be best suited to the emergency department, especially if this is done with existing staff. It seems likely that the opt-in strategy with consent contributed to the low overall screening rate. As a matter of fact, our screening rate of 3.9% is within the range of that observed in other opt-in studies conducted without dedicated staff (average 5.0%). As shown in table 3, modified from Haukoos [28], published screening rates depend both on whether dedicated staff were available (17.4% with and 6.7% without dedicated staff, p<0.001) and on whether an opt-in or op-out approach was used (8.9% versus 27.9%, respectively, p<0.001). So the difference in health care systems does not appear to modify that much the screening rate in EDs.

It should be noted that similar screening rates do not imply similar rates of test proposal, acceptance and uptake. In our study, although the test proposal rate was relatively low (6.2% overall, 2.6% to 10.7% in the different centres), the acceptance rate was high (69.6%), as was the effective test rate (90.9%). D’Almeida et al [26] reported similar rates (acceptance 63.1%, realization 96.4%), in another study conducted in EDs in the Paris area. These results show the good acceptability of HIV screening with rapid tests in the ED setting. The test used here, which provides the result within 5 minutes and gives a limited number of false-positive and invalid results, probably contributed to the high realization rate although no additional staff was provided.

The test proposal rate varied widely across the six centres, probably depending on staff members’ conviction of the utility of HIV screening, and on work organization in each centre. In centre A, which had the lowest proposal rate, there was a conflict among staff members during the first 6 months of the study. The proposal rate diminished over time in all six centres, probably because the ED teams were not convinced of the program’s utility, and the lack of reinforcement during the study. Although the prevalence of new diagnoses was well above the reported cost-effectiveness threshold of 0.1% in each centre, the absolute number in each centre was small, ranging from 5 to 17 (only one centre had more than one new diagnosis per month), making it difficult for the ED staff to realize that the strategy was in fact cost-effective in their local setting. This may well be a critical point when expanding HIV screening in health care settings with limited number of visits per year. It would therefore be important to highlight this notion during training sessions prior to implementing HIV screening.

We found that the proposal rate was influenced by age and gender. Men and younger persons were more likely to be offered the test than were women and older persons. Even though the test was to be proposed systematically, the ED staff implicitly selected the persons to whom they offered the test, based on their own beliefs. This probably explains why the positivity rate was not significantly associated with age. Because this study was designed to resemble real-life screening conditions, only age and sex could be used to evaluate whether persons who were offered the test were implicitly selected by the ED staff. No systematic assessment of the reasons for offering the test was made.

The estimated overall prevalence of previously undiagnosed HIV infection among effectively tested persons was 0.61% (95% CI: 0.46%–0.79%), which is similar to that observed in free anonymous testing centres in the Paris area (0.6%) [14]. It is unclear why it was higher than in the study conducted by D’Almeida et al [26] in EDs in the same region. Possibly, the higher test proposal rate in the latter study (26.7% versus 6.2%) might have been associated with a lower prevalence of previously undiagnosed HIV infection. This would occur if patients with some perceptions of increased risk for HIV were more likely to be approached for consent and also to consent. However, in our study, while the proposal rate fell significantly between the first 3 months and the last 3 months of the study, the positivity rate was not associated with the period (Figure 3), and was not therefore associated with the proposal rate. Likewise, the non-parametric correlation coefficient between the screening and prevalence rates estimated for the studies listed in table 3 was only −0.39 (p = 0.15). Therefore, the most likely explanation for the difference between the two studies is that the 6 centres participating in our study cover populations with different prevalence rates of previously undiagnosed HIV infection than the centres participating in the other study.

As expected, among the newly diagnosed persons, the proportions of MSM and heterosexuals from sub-Saharan Africa were similar to those estimated from incidence data [29], [30]. Among the newly HIV-diagnosed persons who were linked to care, the estimated median CD4 cell count at diagnosis was 241/mm^3^, close to that reported by D’Almeida et al (212/mm^3^). It is noteworthy that 60% of persons diagnosed late in the infection (with AIDS or a CD4 cell count <350/mm^3^) had never been tested before, highlighting the fact that expanded HIV screening would be helpful for patients who have been infected for a long time.

Linkage to care was a specific focus of this study. In each centre the study teams included the staff of the ED, the virology laboratory and the infectious diseases or internal medicine departments, in order to ensure continuity of care for the newly diagnosed persons. The rate of initial linkage to care (87%) was comparable to that reported elsewhere (higher than two-thirds in most studies), even though HIV infection healthcare costs are fully covered by social security. As we have already shown that the risk of being lost to follow-up after initial linkage to care is much higher during the first year following diagnosis than subsequently [31], we followed the newly identified HIV-infected persons for 6 months after initial linkage and found that two-thirds of them remained linked to care. As most studies only report the initial linkage rate, we are unable to compare this figure, but we feel it is important to report not only the initial rate but also the retention rate, after several months, when evaluating routine or expanded HIV screening.

To unmask the hidden HIV epidemic in industrialized countries, there is a need to diversify screening sites and modalities. Non-targeted general population-based strategies likely complement targeted community-based strategies. This study shows that, because of its good acceptability and cost-effectiveness (undiagnosed HIV prevalence well above 0.1%), HIV screening in emergency departments may be recommended in areas of France with a high prevalence of HIV infection.

## Supporting Information

Table S1 **Results of a 12 month routine HIV screening program with a rapid test after written inform consent in non eligible patients at 6 university hospital emergency departments (EDs) in Paris area (2009–2011).**
(DOC)
