# Supplementary material for: Twelve Months of Routine HIV Screening in 6 Emergency Departments in the Paris Area: Results from the ANRS URDEP Study
Source: PLoS One. 2012 Oct 2;7(10):e46437. doi: 10.1371/journal.pone.0046437 (PMC3462802; doi:10.1371/journal.pone.0046437)
Supplement: Table S1 — Results of a 12 month routine HIV screening program with a rapid test after written inform consent in non eligible patients at 6 university hospital emergency departments (EDs) in Paris area (2009–2011). (DOC) [file pone.0046437.s001.doc]

**Table S1 Results of a 12 month routine HIV screening program with a rapid test after written inform consent in non eligible patients at 6 university hospital emergency departments (EDs) in Paris area (2009-2011)**

| **Centre** | **A** | **B** | **C** | **D** | **E** | **F** | **TOTAL** |
| --- | --- | --- | --- | --- | --- | --- | --- |
| **Persons examined at EDs** | 51111 | 44611 | 68234 | 42525 | 68511 | 36161 | 311153 |
| **Non eligible persons** | 20827 | 19739 | 40052 | 14026 | 23333 | 9219 | 127196 |
| **Offered HIV testing** | 275 | 414 | 222 | 944 | 552 | 213 | 2520 |
|  | (0.8%) | (2.1%) | (0.6%) | (6.7%) | (2.4%) | (2.3%) | (2.0%) |
| **Accepting HIV testing** | 150 | 350 | 197 | 710 | 474 | 182 | 2063 |
|  | (85.7%) | (84.5%) | (88.7%) | (75.2%) | (85.9%) | (85.4%) | (81.9%) |
| **Tested for HIV** | 140 | 285 | 186 | 599 | 466 | 181 | 1857 |
|  | (93.3%) | (81.4%) | (94.4%) | (84.4%) | (98.3%) | (99.5%) | (90.0%) |
| **Rate tested for HIV among ED patients** | 0.7% | 1.4% | 0.5% | 4.3% | 2.0% | 2.0% | 1.5% |
| **Positive rapid test** | 0 | 6 | 1 | 7 | 9 | 0 | 23 |
| **Positive test in previously unknown HIV-infected patients** | 0 | 4 | 1 | 6 | 9 | 0 | 20 |
| **Western-blot confirmatory test performed** | 0 | 4 | 1 | 3 | 7 | 0 | 15 |
|  | - | (100%) | (100%) | (50%) | (78%) | - | (81%) |
| **Newly identified HIV infected patients** | 0 | 4 | 1 | 3 | 7 | 0 | 15 |
| **Rate of newly identified HIV-infected patients among tested eligible patients** | 0.00% | 1.40% | 0.54% | 0.50% | 1.50% | 0.00% | 0.81% |
| **Linked to care** | 0 | 3 | 0 | 3 | 5 | 0 | 11 |
|  | - | (75%) | (0%) | (100%) | (71%) | - | (73%) |
| **Not lost to follow-up at month 6** | 0 | 2 | 0 | 2 | 4 | 0 | 8 |
|  | - | (67%) | (0%) | (67%) | (80%) | - | (73%) |
